# Validity and Prognostic Value of a Polygenic Risk Score for Parkinson’s Disease

**DOI:** 10.3390/genes12121859

**Published:** 2021-11-23

**Authors:** Sebastian Koch, Björn-Hergen Laabs, Meike Kasten, Eva-Juliane Vollstedt, Jos Becktepe, Norbert Brüggemann, Andre Franke, Ulrike M. Krämer, Gregor Kuhlenbäumer, Wolfgang Lieb, Brit Mollenhauer, Miriam Neis, Claudia Trenkwalder, Eva Schäffer, Tatiana Usnich, Michael Wittig, Christine Klein, Inke R. König, Katja Lohmann, Michael Krawczak, Amke Caliebe

**Affiliations:** 1 Institute of Medical Informatics and Statistics, Kiel University, University Medical Center Schleswig-Holstein, Campus Kiel, 24105 Kiel, Germany; koch@medinfo.uni-kiel.de (S.K.); krawczak@medinfo.uni-kiel.de (M.K.); 2 Institute of Medical Biometry and Statistics, University of Luebeck, University Medical Center Schleswig-Holstein, Campus Luebeck, 23562 Luebeck, Germany; b.laabs@uni-luebeck.de (B.-H.L.); inke.koenig@uni-luebeck.de (I.R.K.); 3 Department of Psychiatry, University of Luebeck, 23538 Luebeck, Germany; meike.kasten@neuro.uni-luebeck.de; 4 Institute of Neurogenetics, University of Luebeck, University Medical Center Schleswig-Holstein, Campus Luebeck, 23538 Luebeck, Germany; jule.vollstedt@neuro.uni-luebeck.de (E.-J.V.); norbert.brueggemann@neuro.uni-luebeck.de (N.B.); tatiana.usnich@neuro.uni-luebeck.de (T.U.); christine.klein@neuro.uni-luebeck.de (C.K.); katja.lohmann@neuro.uni-luebeck.de (K.L.); 5 Department of Neurology, Kiel University, 24105 Kiel, Germany; jossteffen.becktepe@uksh.de (J.B.); g.kuhlenbaeumer@neurologie.uni-kiel.de (G.K.); eva.schaeffer@uksh.de (E.S.); 6 Department of Neurology, University of Luebeck, 23562 Luebeck, Germany; ulrike.kraemer@neuro.uni-luebeck.de (U.M.K.); mi.neis@uni-luebeck.de (M.N.); 7 Institute of Clinical Molecular Biology, Kiel University, 24105 Kiel, Germany; a.franke@mucosa.de (A.F.); m.wittig@mucosa.de (M.W.); 8 Institute of Epidemiology and PopGen Biobank, Kiel University, University Medical Center Schleswig-Holstein, Campus Kiel, 24105 Kiel, Germany; wolfgang.lieb@epi.uni-kiel.de; 9 Department of Neurology, University Medical Center Goettingen, 37075 Goettingen, Germany; brit.mollenhauer@med.uni-goettingen.de; 10 Paracelsus-Elena-Klinik, 34128 Kassel, Germany; ctrenkwalder@gmx.de; 11 Department of Midwifery Science, University of Luebeck, 23562 Luebeck, Germany; 12 Department of Neurosurgery, University Medical Center Goettingen, 37075 Goettingen, Germany

**Keywords:** Parkinson’s disease, polygenic risk score, replication, validation, prognostic value, genetic risk

## Abstract

Idiopathic Parkinson’s disease (PD) is a complex multifactorial disorder caused by the interplay of both genetic and non-genetic risk factors. Polygenic risk scores (PRSs) are one way to aggregate the effects of a large number of genetic variants upon the risk for a disease like PD in a single quantity. However, reassessment of the performance of a given PRS in independent data sets is a precondition for establishing the PRS as a valid tool to this end. We studied a previously proposed PRS for PD in a separate genetic data set, comprising 1914 PD cases and 4464 controls, and were able to replicate its ability to differentiate between cases and controls. We also assessed theoretically the prognostic value of the PD-PRS, i.e., its ability to predict the development of PD in later life for healthy individuals. As it turned out, the PD-PRS alone can be expected to perform poorly in this regard. Therefore, we conclude that the PD-PRS could serve as an important research tool, but that meaningful PRS-based prognosis of PD at an individual level is not feasible.

## 1. Introduction

Parkinson’s disease (PD) is the second most common neurodegenerative disorder after Alzheimer’s disease, with a particularly high prevalence seen in Europe and North America [1]. PD has a complex multifactorial etiology in which both environmental and genetic factors play a prominent role. The main risk factor for PD hitherto identified, however, is age, and both prevalence and incidence increase exponentially in later life.

While some 3–5% of PD cases are monogenic, recent genome-wide association studies (GWAS) revealed that idiopathic PD is highly polygenic [2,3,4]. Therefore, the development of polygenic risk scores (PRSs) as a means to summarize the effect of the genetic background upon an individual’s disease risk in a single number appears meaningful for idiopathic PD. Several PRSs have been developed for PD affection status, age-at-onset and specific symptoms in studies of variable size and using different methodologies [2,5,6,7,8,9,10].

Although the construction of a PRS is rather straightforward using existing software, the validation of existing PRSs through an assessment of their performance in independent data sets has still been undertaken only rarely and, to our knowledge, not for PD. One aim of our study therefore was to investigate in more detail the discriminatory power of a PRS for PD previously published by Nalls et al. [2]. This PRS was developed based upon the largest meta-GWAS for the disease to date and comprises 1805 single nucleotide polymorphisms (SNPs). Our second aim was to assess the prognostic value of this PD-PRS. In fact, while PRSs usually differentiate well between cases and controls, their utility for disease prognostics has been a matter of intensive debate [11,12].

## 2. Materials and Methods

### 2.1. Samples

The samples analyzed in the present study originated from five German cohorts comprising a total of 1914 PD cases and 4464 controls after quality control (Table A1). The data sets were collated within the framework of DFG Research Unit ’ProtectMove’ (FOR2488). The samples of two PD patient and control cohorts (Kiel PD, Luebeck PD) were recruited locally in Schleswig-Holstein, the northernmost federal state of Germany. EPIPARK is an additional prospective and longitudinal observational single-center study from Luebeck, focused upon the non-motor symptoms of PD patients [13]. DeNoPa is a prospective and longitudinal observational single-center study from Kassel in central Germany, aimed specifically at improving early diagnosis and prognosis of PD. Participants include early untreated PD patients and matched healthy controls [14]. The PopGen biobank [15,16] is a central research infrastructure, maintained by Kiel University, for the recruitment of case-control cohorts for defined diseases [15,16]. For the present study, PopGen contributed 661 PD patients and 3093 unaffected individuals from the broader Kiel area.

### 2.2. Genotyping, Genotype Imputation and Quality Control

Genomic DNA was extracted from peripheral blood leukocytes and genotyped using the Infinium Global Screening Array with Custom Content (GSA; Illumina Inc., San Diego, CA, USA) which targets 645,896 variants. Quality control was performed with PLINK 1.9, PLINK 2.0 and R package *plinkQC* [17,18,19,20,21,22]. 

At the SNP level, quality control was carried out with thresholds of 0.01 for the minor allele frequency (MAF), of 0.98 for the SNP call rate and of 10^−50^ for the software-issued p value of the Hardy–Weinberg equilibrium test. Some 431,738 variants passed quality control and were used for imputation with *SHAPEIT2* [23] and *IMPUTE2* [24], based upon the public part of the HRC reference panel (release 1.1, The European Genome-Phenome Archive, EGAS00001001710) [25]. Imputation yielded genotype data for a total of 39,106,911 variants and after the exclusion of variants with MAF < 0.01 or an info score < 0.7, some 7,804,284 variants remained for further analyses.

**Figure A1:**
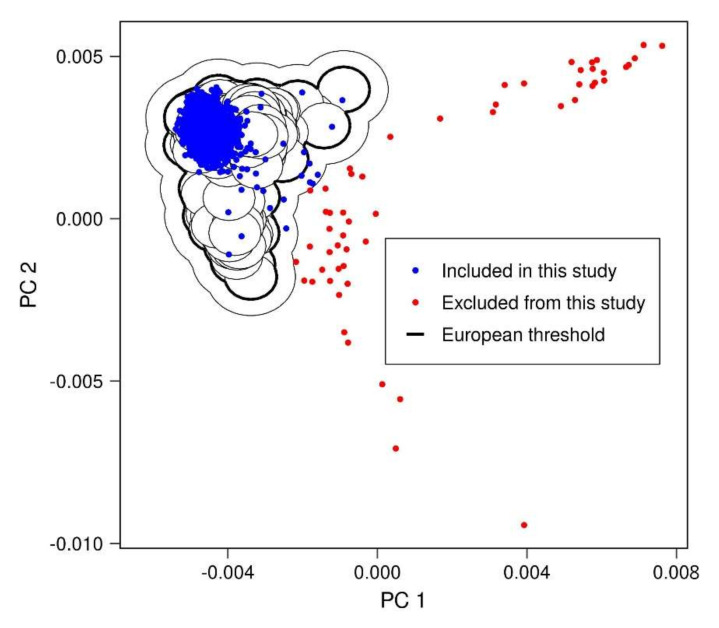
Identification of population outliers by PCA drawing upon 1000Genomes data. White circles represent polygonal circle approximations around European samples of the 1000Genomes project. The thick black line marks the union set, the thinner line marks the final boundary. Dots representing our samples are colored according to their inclusion in or exclusion from the study. Samples were excluded if they were outside the boundary. PC: principal component, PCA: principal component analysis.

**Figure A2:**
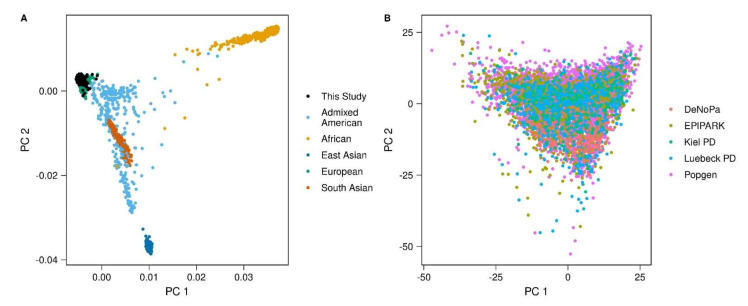
PCA plots after quality control. (**A**) Plot of the first two PCs from the 1000Genomes supra populations and the samples of this study. Our study samples were plotted on top, therefore obscuring part of the European samples from the 1000Genomes project. (**B**) Plot of the first two PCs from the cohorts included in our study (Table A1). PC: principal component, PCA: principal component analysis.

At the participant level, 6794 individuals were initially available from the five cohorts. Individuals with a call rate < 0.98 or with a heterozygosity value > 3 standard deviations different from the mean on the non-imputed data were removed. To exclude potential relatives and population outliers, linkage disequilibrium pruning was performed using a window size of 50 variants, shifted by five variants, and an r^2^ threshold of 0.2, leaving 186,064 variants. Pairwise identity-by-descent (IBD) was then estimated and individuals were removed in a customized selection process (see Appendix A.1) until all pairwise IBD values were <0.1. For details on the identification of population outliers, see Appendix A.2 and Figure A1. In total, 416 individuals were removed leaving 6378 individuals (1914 cases, 4464 controls) for further analysis. Principal component analysis (PCA) plots of the samples from our study and from the 1000Genomes project can be found in Figure A2.

### 2.3. Analysis of Parkinson’s Disease Polygenic Risk Score (PD-PRS)

We evaluated a PRS for PD published by Nalls et al. [2]. The list of the 1805 SNPs included in this PD-PRS, together with reference alleles and effect sizes, was kindly provided to us by the first author. Matching the SNPs to our imputed SNPs was done by reference to their chromosomal positions. Some 1743 of the PD-PRS SNPs were represented in our data set, and all of these SNPs were imputed (the 62 omitted SNPs are listed in Table A2). 

The PD-PRS values were standardized by subtraction of the mean and division by the standard deviation of the PD-PRS among controls. This standardized version of the PRS will henceforth be used and also referred to as ‘PD-PRS’ as well. Density plots were created with base-R function *density*. Logistic regression analysis was performed treating the case-control status as outcome and the PD-PRS value as influence variable, adjusted for the first three PCs, sex and age-at-sampling. An additional logistic regression analysis, excluding age-at-sampling, was performed among cases from the lowest and highest age-at-onset quartiles, treating quartile affiliation as outcome. A two-sided significance level of 0.05 was adopted for the Wald test embedded into the logistic regression analysis.

Receiver operating characteristic (ROC) curves and corresponding areas under curve (AUCs) were calculated with R package *pROC* [26] and 95% confidence intervals for odds ratios were constructed with the *oddsratio.wald* function from the *epitools* package [27].

### 2.4. Identification of Most Relevant PD-PRS SNPs

We evaluated which SNPs of the PD-PRS were most relevant for distinguishing cases from controls by determining their influence upon the AUC. This was done in three steps.

1. The PD-PRS was repeatedly calculated, excluding one SNP each time, and determining the AUC of the PD-PRS without the SNP. These AUCs will be referred to as ‘AUC-SNP’ values.
2. SNPs were sequentially removed from the PD-PRS based upon the steepest decline of the AUC of the remaining SNPs, until the 95% confidence interval of the residual AUC included 0.5. This set of removed SNPs will be referred to as ‘most relevant SNPs’.
3. The results from step 1 and step 2 were combined in a single plot, relating the AUC-SNP values of SNPs (y axis) to their AUC-SNP-based rank (x axis) and color-coding the set of most relevant SNPs from step 2 together with the set of 47 genome-wide significant SNPs identified by Nalls et al. [2] and included in our PD-PRS.

R package *biomaRt* and the hsapiens_gene_ensembl data set from Ensembl were used to identify genes that included at least one of the most relevant SNPs [28,29,30]. Coding and functional information on individual SNPs were obtained from dbSNP [31].

### 2.5. Prognostic Value of PD-PRS

The *coords* function from R package *pROC* [26] was used to derive appropriate PD-PRS thresholds from ROC curves, and to determine the corresponding values of sensitivity and specificity. Thresholds were calculated by maximizing a weighted Youden-Index:

max(costs ∙ sensitivity + specificity)

where ‘costs’ was defined as the relative severity of a false negative compared to a false positive result (i.e., classification or prediction as PD). Costs were varied from 1 to 5 in steps of 0.0001.

For fixed specificity and sensitivity, the positive and negative predictive values (ppv, npv) were computed with Bayes formula as

$$ppv=\frac{sensitivity\cdot prevalence}{sensitivity\cdot prevalence+\left(1-specificity\right)\cdot \left(1-prevalence\right)}$$

$$npv=\frac{specificity\cdot \left(1-prevalence\right)}{specificity\cdot \left(1-prevalence\right)+\left(1-sensitivity\right)\cdot prevalence}$$

To evaluate the prognostic value of the PD-PRS, we had to include the residual lifetime incidence in the above formulae instead of the disease prevalence. To this end, we adopted the age-specific incidence and death rates *I*_[interval]_ and *D*_[interval]_ from the SIa strategy in [32]. The SIa strategy used only cases with at least two diagnoses of PD to avoid false positive diagnoses. *I*_[interval]_ and *D*_[interval]_ were given for 5-year age intervals, starting from [50–54] and ending with [95+]. Since the death rates were given as annual probabilities to die within a given interval, the probability to survive that interval can be approximated by *S*_[interval]_ = (1 − *D*_[interval]_)^5^. For individuals from a given age interval [d,d+5], the residual lifetime incidence can then be computed as

*I*_[d, 95+]_ = *I*_[d, d+5]_ + (*I*_[d+6, d+11]_*∙S*_[d, d+5]_∙(1 − *I*_[d, d+5]_)) + … *+* (*I*_[95+]_*∙S*_[d, d+5]_∙…∙*S*_[90, 94]_∙(1 − *I*_[d, d+5]_)∙ … ∙(1-*I*_[90, 94]_)).

The resulting residual lifetime incidence values are listed in Table A3.

## 3. Results

### 3.1. Validation of Published Parkinson’s Disease Polygenic Risk Score (PD-PRS)

**Figure 1:**
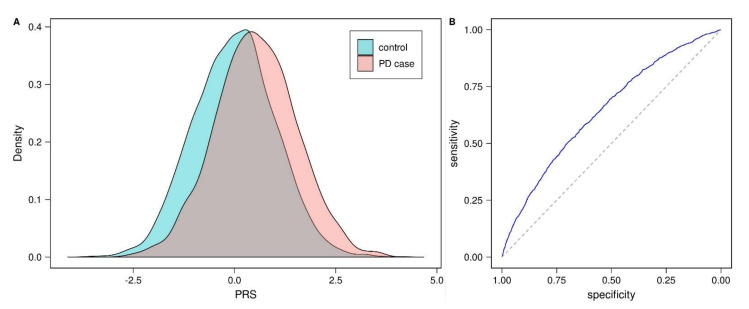
PD-PRS in PD cases and controls. (**A**) Density of PD-PRS in cases and controls. (**B**) ROC curve for PD-PRS as a predictor of case-control status. PRS: polygenic risk score, PD: Parkinson’s disease, ROC: receiver operating characteristic.

**Figure 2:**
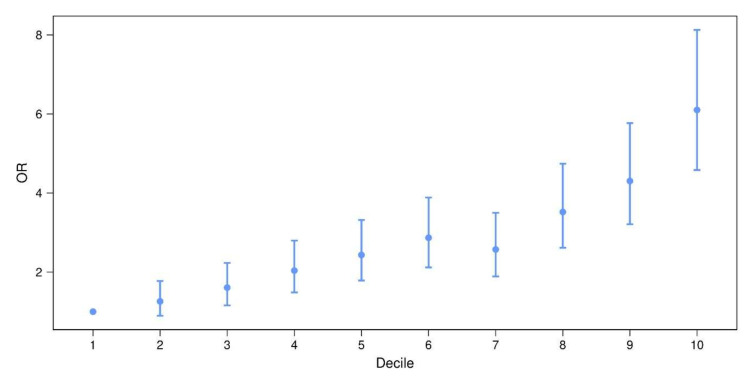
Disease OR for the 2nd to 10th deciles of the PD-PRS distribution among controls. (1st decile used as reference). Vertical bars demarcate 95% confidence intervals. OR: odds ratio, PD: Parkinson’s disease, PRS: polygenic risk score.

To independently validate the (standardized) PD-PRS proposed by Nalls et al. [2], we investigated the performance of this PRS in a separate data set comprising 1914 PD cases and 4464 controls (Table A1). The distribution of the PD-PRS clearly differed between the two groups (Figure 1A; Wald test *p* < 10^−5^, Table 1). Nagelkerke’s pseudo-R^2^ from the logistic regression analysis equaled 0.35 when including PD-PRS, sex, age and the first three principal components (PCs), and 0.30 when the PD-PRS was not included (Table 1). The area under curve (AUC) for the receiver operating characteristic (ROC) curve (Figure 1B) was 0.65, which was comparable to the AUC obtained in the original study [2]. The disease odds ratios (ORs) for the 2nd to 10th deciles of the PRS distribution among controls ranged from 1.26 (2nd decile) to 6.10 (10th decile; 1st decile used as reference; Figure 2).

**Figure 3:**
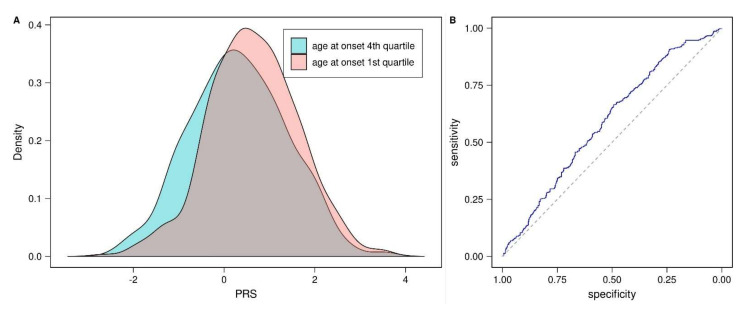
PD-PRS in early and late onset cases. (**A**) Density of PD-PRS in the 1st and 4th AAO quartile of cases. (**B**) ROC curve for PD-PRS as a predictor of 1st vs 4th AAO quartile. AAO: age-at-onset, PRS: polygenic risk score, PD: Parkinson’s disease, ROC: receiver operating characteristic.

The PD-PRS was also able to distinguish well between cases from the 1st and 4th age-at-onset (AAO) quartile (≤54 years vs. >70 years, Figure 3A, *p* = 1.61 × 10^−5^, Table 1). Nagelkerke’s pseudo-R^2^ from the logistic regression was 0.039 including PD-PRS, sex and the first three PCs, and 0.009 when the PD-PRS was not included. The AUC of the ROC equaled 0.59 (Figure 3B, Table 1) and was hence considerably smaller than the AUC obtained for distinguishing cases from controls.

### 3.2. Most Relevant SNPs in PD-PRS

**Figure 4:**
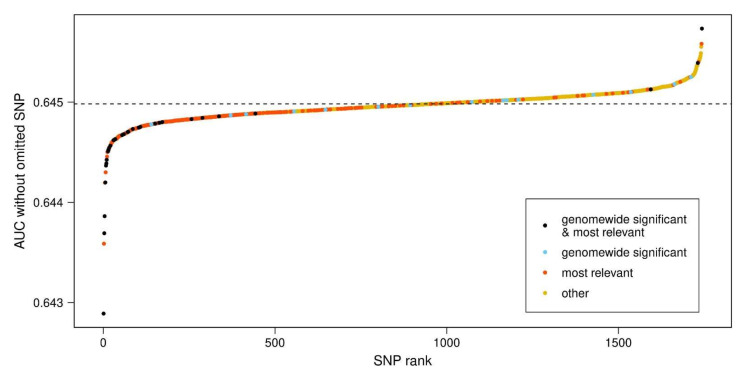
Influence of individual SNPs upon PD-PRS performance. For each of the 1743 PD-PRS SNPs, the AUC was calculated after removing the SNP from the PRS. SNPs were color-coded as either genome-wide significant in a meta-GWAS [2] (blue), as ‘most relevant’ in the present study (red), both of the former (black) or none of the former (yellow). SNP: single nucleotide polymorphism, PD: Parkinson’s disease, PRS: polygenic risk score, AUC: area under ROC curve, ROC: receiver operating characteristic, GWAS: genome-wide association study.

We identified 422 SNPs as being the most relevant for distinguishing cases from controls, judged by their influence upon the AUC in a backward-selection process (see Methods). Of these SNPs, 287 are located within a gene. Table 2 lists the top 20 most relevant SNPs inside genes (for a complete list, see Table A4). Of all 1743 SNPs analyzed, some 47 had been genome-wide significant in the meta-GWAS by Nalls et al. [2]. Thirty-two of these (68%) were among the 422 most relevant SNPs identified here, and 25 of them (78%) were intra-genic. When all 1743 SNPs were ranked according to the AUC obtained when a given SNP was removed (Figure 4), the 422 most relevant SNPs occurred mostly on the left side of the graph meaning that the AUC is strongly reduced upon the removal of the SNP. The 32 most relevant and genome-wide significant SNPs, in particular, were found to cluster at the far left of the graph.

### 3.3. Prognostic Value of PD-PRS

**Figure 5:**
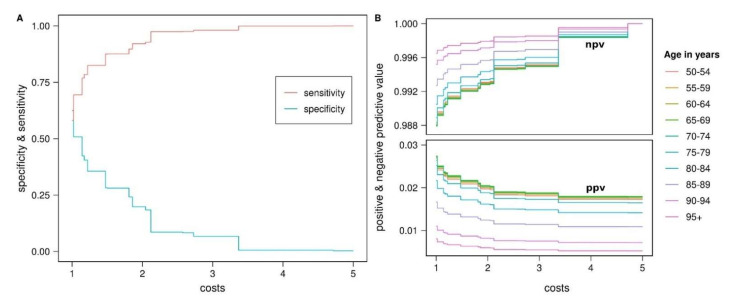
Prognostic value of PD-PRS. (**A**) Sensitivity and specificity of PD-PRS for the optimal threshold were determined by maximizing a weighted Youden index. The relative costs of false negative vs false positive results varied from 1 to 5. (**B**) ppv and npv were calculated from the costs-based sensitivity and specificity and the residual lifetime incidence (see Methods and Table A3) in 10 age groups. PRS: polygenic risk score, PD: Parkinson’s disease, ppv: positive predictive value, npv: negative predictive value.

To investigate the prognostic value of the PD-PRS, an individual was defined as ‘test-positive’ if their PRS exceeded a given threshold of the PRS and ‘test-negative’ if not. Thus, sensitivity in this context means the probability that a person who develops PD in later life has a PRS above the threshold while specificity is the probability that a person who will not develop PD during their lifetime is test-negative. Since sensitivity is generally more important than specificity for screening tests, we considered different relative costs of false negative vs false positive test results when maximizing a weighted Youden index to determine the optimal PD-PRS threshold (Table 3). For costs of 1, i.e., when false positives and false negatives are deemed equally serious, the optimal PD-PRS threshold equaled 0.33, yielding a sensitivity of 0.58 and a specificity of 0.63. For costs of 5, the sensitivity equaled 1 and the specificity equaled 0.003 at an optimal PD-PRS threshold of −2.667 (Table 3, Figure 5A).

For fixed costs, the age-specific predictive values of the PD-PRS differed only little up to age interval [70–74], after which the positive predictive value (ppv) declined and the negative predictive value (npv) increased (Table 4, Figure 5B). Across all age groups and costs levels, the ppv was very low with a maximum of 0.027 up to 74 years at costs of 1. The minimum ppv was 0.005 for the highest age group (90+) at costs of 5. The npv varied between 0.988 (≤74 years, costs 1) and 1 (all age groups, costs 5).

## 4. Discussion

In the present study, we replicated the performance of the PD-PRS developed by Nalls et al. [2] in an independent data set. It turned out that the PD-PRS was clearly able to distinguish between cases and controls and that it was increased in cases of early age-at-onset. Individuals in the 10th PRS decile had an OR of around 6 of having PD as compared to individuals in the lowest decile. This is in line with the results by Nalls et al. [2] who reported ORs of 3.74 and 6.25 for the highest quartiles in their two data sets. The most relevant PRS SNPs identified in our study included many genome-wide significant SNPs from the Nalls et al. study [2], as was to be expected. In fact, of the 47 genome-wide significant SNPs, some 32 (68%) were found to be most relevant in the sense of our study. However, this is still only a small fraction (7.5%) of the total number of 422 most relevant SNPs, which highlights the polygenic background of PD with several low-effect variants and justifies the fact that not only genome-wide significant SNPs were originally included in the PRS.

In the recent past, the research community has become increasingly aware of the problem of non-replicability of research findings in independent data sets or with different methods [33]. This has been termed the “replication crisis” or “reproducibility crisis” [34,35]. Studies aiming at validating existing PRSs are still rare and, usually, new data set-specific PRSs are developed instead because this is easy with existing software. Nevertheless, PRS replication should be mandatory [36] and our replication of the results reported by Nalls et al. [2], in an independent data set, is reassuring. It supports the idea that this PD-PRS can be used to capture the contribution of the genetic background of an individual to their PD risk. The PD-PRS could hence be a valid instrument to adjust for the genetic background component in statistical models for PD. Moreover, it may also facilitate studies of the genetic overlap between different diseases or disease subtypes and of the interaction between genetic and environmental factors.

It has to be kept in mind, however, that PRSs only capture the effect of common genetic variants. Highly-penetrant rare or private variants as well as other types of variations such as copy number variants or indels are not represented [37]. Another drawback of PRSs is their dependency on the ancestry of populations [38]. The PD-PRS analyzed in the present study was both constructed and validated in populations of European ancestry, and transferability of the results to other ancestries cannot be taken for granted but has to be investigated in future studies. On a related note, it must be kept in mind that all PD-PRS SNPs considered in our study were imputed. This does not seem to have impaired our replication of the results of Nalls et al. [2], probably due to our stringent quality control. For populations, where a good imputation reference is lacking, consistent PRS performance may not be taken for granted.

Quality control in our study led to the exclusion of 62 of the original 1805 PD-PRS SNPs. The omitted SNPs showed on average a larger effect size in the original meta-analysis than the SNPs included in our PRS (Table A2). The former were excluded mostly (79%) because of very low MAF and the rest because the info score was below 0.70. Despite the higher effect sizes, it is therefore not clear if the additional usage of the 62 SNPs would enhance the performance of the PD-PRS because of low MAF and perhaps difficult imputation. The loss of variants from the score due to difficulties in imputation is a good argument for the adoption of the development of standardized PRSs based on reference variants which are available in common genotyping arrays. This would reduce the imputation problem. 

Whereas PRSs deserve a role in etiological research and statistical modelling of diseases, their prognostic value is dubious [11,12,36]. PRSs are developed to differentiate between cases and controls. Although the level of differentiation achieved is reasonable at a group level, the obtained AUCs are usually insufficient for individual diagnostic or prognostic testing, where an AUC > 0.90 is required [11]. In this study, we evaluated the prognostic value of a specific PD-PRS and calculated its sensitivity and specificity as well as its predictive values for various assumptions about the relative importance of mis-prognoses. Our results were in accordance with the generally held view that a prognostic application of PRSs alone is not meaningful. The negative predictive values were high which means that people with a low PRS can be reasonably sure not to develop PD, at least not of the type considered in this study. However, the positive predictive values were only of the order of a few percent which means that the probability of a person with a high PRS developing the disease is quite low. Here, the comparison to a hypothetical test which gives everybody a negative test result is helpful: Assuming a lifetime incidence of 5% [39], the negative predictive value of this (nonsense) test would be 95%, i.e., quite similar to a test based solely on the PD-PRS.

There are three ways in which a prognostic test for PD, or any other disease, could potentially help to reduce incidence or severity: change of lifestyle factors, enhanced surveillance or preventive treatment. Of these, a change towards a healthier lifestyle is always meaningful, both from an individual and a population health perspective, and only a test with a positive predictive value much higher, for example, than that of the PD-PRS would mean an additional individual incentive for change. Moreover, with a low incidence and positive predictive value, frequent medical screening of individuals with a high PRS would mean spending valuable resources for individuals who have only a probability of a few percent to actually develop the disease in question. The same holds true for possible preventive treatment if such treatment were available in the first place. Apart from economic constraints, side-effects might result in a negative benefit-risk balance when the incidence of the disease in question is as low as for PD.

A limitation of our study has been that the predictive values were only calculated from theoretical models and were not based directly upon empirical observations. This is a general drawback when evaluating the prognostic value of PRSs because adequate long-term studies would be time-consuming, require large sample sizes and would hence be rather expensive. This notwithstanding, PRSs have to be externally validated and compared to other (clinical) risk models in a clinically meaningful prospective set-up [12,36] because this is a *conditio sine qua non* for the applicability in practice of any prognostic marker. Only a few studies have taken first steps in this direction [40,41,42], and most have found none or only little additional prognostic value of PRSs over and above clinical and demographic predictors. To our knowledge, no such study has been performed yet for PD, where the combination of a PRS with established prodromal markers [43] might be specifically worth investigating in future prospective studies.

## 5. Conclusions

The PD-PRS proposed by Nalls et al. [2] could be validated independently in German patients and controls, suggesting that the PRS may be a meaningful research tool to investigate and adjust for the polygenic component of PD. Individual risk prediction using the PD-PRS alone is, however, not meaningful.

## Figures and Tables

**Table 1:** Comparative validation of PD-PRS.

Data Set	Samples (N)	SNPs (N)	AUC [95% CI]	Nagelkerke’s Pseudo-R^2 a^	*p* Value ^b^	Nagelkerke’s Pseudo-R^2 c^
This study (case/control)	6378	1743	0.645 [0.630, 0.660]	0.348	<10^−5^	0.298
Nalls training ^d^ (case/control)	11,243	1809	0.640 [0.630, 0.650]	n.a.	<10^−5^	n.a.
Nalls validation ^e^ (case/control)	999	1805	0.692 [0.660, 0.725]	n.a.	<10^−5^	n.a.
This study (AAO) ^f^	836	1743	0.590 [0.551, 0.629]	0.039	1.6 × 10^−5^	0.009

^a^ From logistic regression analysis of PD case-control status (first line) and AAO 1st vs 4th quartile (fourth line), each time including PD-PRS, sex, age (only for the analysis of case-control status) and the first three PCs as independent variables. Nalls et al. [2] used a different approach to evaluate logistic regression models, hence a comparison of pseudo-R^2^ is not meaningful. ^b^ *p* value for PD-PRS as an independent variable in the logistic regression analysis (Wald test). ^c^ Same logistic regression model as before, but without PD-PRS as an independent variable. ^d^ NeuroX-dbGaP data set (5851 cases, 5866 controls). ^e^ Harvard Biomarker Study (527 cases, 472 controls). ^f^ Samples belonging to the 1st and 4th AAO quartile among cases analyzed in this study. PD: Parkinson’s disease, PRS: polygenic risk score, SNP: single nucleotide polymorphism, AUC: area under ROC curve, CI: confidence interval, AAO: age-at-onset, ROC: receiver operating characteristic, n.a.: not available.

**Table 2:** Top 20 most relevant SNPs located within genes.

HGNC Symbol ^1^	Chr	AUC	Start ^2^	End ^3^	SNP Position ^4^	A1 ^5^	A2 ^6^	GS ^7^	SNP Type
ENSG00000251095	4	0.643	90,472,507	90,647,654	90,626,111	G	A	yes	intron
*SNCA*	4	0.641	90,645,250	90,759,466	90,684,278	A	G	no	intron
*HIP1R*	12	0.640	123,319,000	123,347,507	123,326,598	G	T	yes	intron
*TMEM175*	4	0.639	926,175	952,444	951,947	T	C	yes	missense
*SNCA*	4	0.638	90,645,250	90,759,466	90,757,294	A	C	no	intron
*ASH1L*	1	0.637	155,305,059	155,532,598	155,437,711	G	A	no	intron
*UBQLN4*	1	0.634	156,005,092	156,023,585	156,007,988	G	A	no	intron
ENSG00000225342	12	0.633	40,579,811	40,617,605	40,614,434	C	T	yes	n.a.
*LRRK2*	12	0.633	40,590,546	40,763,087	40,614,434	C	T	yes	n.a.
*STX1B*	16	0.632	31,000,577	31,021,949	31,004,169	T	C	no	synonymous
*INPP5F*	10	0.631	121,485,609	121,588,652	121,536,327	G	A	yes	intron
*CCSER1*	4	0.631	91,048,686	92,523,064	91,164,040	C	T	no	intron
*SLC2A13*	12	0.630	40,148,823	40,499,891	40,388,109	C	T	no	intron
*FBXL19*	16	0.630	30,934,376	30,960,104	30,943,096	A	G	no	intron
ENSG00000251095	4	0.629	90,472,507	90,647,654	90,619,032	C	T	no	intron
*CAB39L*	13	0.629	49,882,786	50,018,262	49,927,732	T	C	yes	intron
*STK39*	2	0.628	168,810,530	169,104,651	168,979,290	C	T	no	intron
*CCT3*	1	0.628	156,278,759	156,337,664	156,300,731	T	C	no	intron
ENSG00000225342	12	0.627	40,579,811	40,617,605	40,614,656	A	G	no	n.a.
*LRRK2*	12	0.627	40,590,546	40,763,087	40,614,656	A	G	no	n.a.

^1^ HGNC symbol or Ensemble gene ID if there is no HGNC symbol available. ^2^ Base pair position of start of gene. ^3^ Base pair position of end of gene. ^4^ Genomic position of SNP. ^5^ Major SNP allele. ^6^ Minor SNP allele. ^7^ Genome-wide significant (GS) in the meta-GWAS by Nalls et al. [2]. HGNC: HUGO Gene Nomenclature Committee, Chr: Chromosome, AUC: area under ROC curve, ROC: receiver operating characteristic, PRS: polygenic risk score, PD: Parkinson’s disease, n.a.: not available.

**Table 3:** Prognostic value of PD-PRS.

	Costs				
	1	2	3	4	5
Sensitivity [95% CI]	0.581 [0.479, 0.733]	0.921 [0.880, 0.981]	0.981 [0.973, 1]	0.999 [0.983, 1]	1 [0.996, 1]
Specificity [95% CI]	0.625 [0.472, 0.725]	0.198 [0.075, 0.289]	0.067 [0.004, 0.096]	0.006 [0.002, 0.082]	0.003 [0.002, 0.034]
Threshold ^1^	0.330	−0.868	−1.507	−2.533	−2.667

^1^ Optimal threshold for PD-PRS as determined by maximizing a weighed Youden index. PD: Parkinson’s disease, PRS: polygenic risk score, CI: confidence interval.

**Table 4:** Costs- and age-dependent PD-PRS predictive values.

		Costs									
		1		2		3		4		5	
		ppv	npv	ppv	npv	ppv	npv	ppv	npv	ppv	npv
**Age group (Years)**	50–54	0.026	0.988	0.020	0.993	0.018	0.995	0.017	0.998	0.017	1
	55–59	0.027	0.988	0.020	0.993	0.018	0.995	0.018	0.998	0.018	1
	60–64	0.027	0.988	0.020	0.993	0.019	0.995	0.018	0.998	0.018	1
	65–69	0.027	0.988	0.021	0.993	0.019	0.995	0.018	0.998	0.018	1
	70–74	0.027	0.988	0.020	0.993	0.019	0.995	0.018	0.998	0.018	1
	75–79	0.025	0.989	0.019	0.993	0.017	0.995	0.017	0.999	0.016	1
	80–84	0.022	0.990	0.016	0.994	0.015	0.996	0.014	0.999	0.014	1
	85–89	0.017	0.993	0.012	0.996	0.011	0.997	0.011	0.999	0.011	1
	90–94	0.011	0.995	0.008	0.997	0.008	0.998	0.007	0.999	0.007	1
	95+	0.008	0.996	0.006	0.998	0.005	0.999	0.005	1.000	0.005	1

PRS: polygenic risk score, PD: Parkinson’s disease, ppv: positive predictive value, npv: negative predictive value.

## Data Availability

The data that support the results of this study are available upon reasonable request from the corresponding author.

## Appendix A

### Appendix A.1. Removal of Related Individuals

Clusters of related individuals were generated such that each individual in a cluster had an IBD value ≥ 0.1 with at least one other individual in the cluster. Typical clusters were siblings or parent-child clusters but also larger clusters of extended families were found. A total of 238 disjunct clusters comprising 503 individuals were detected in our data set. For each cluster, the largest subset of unrelated individuals (all pairwise IBD values < 0.1) was next selected, and since cases were more valuable for our analysis than controls, the former were given double weight in the selection process. If two equally large subsets remained, the subset with the highest AAO for a case was selected because idiopathic PD typically has high AAO. If this was not possible, selection was in favor of the subset with the oldest control. Of the 503 individuals in clusters, 243 were kept for further analysis.

### Appendix A.2. Removal of Population Outliers

Population outliers were removed in our study by two different approaches. In the first approach, our data set was merged with 2504 individuals from the 1000Genomes project (1000 Genomes Phase III, imputed). A PCA was then done with *PLINK 1.9* [21] at the default setting of 20 PCs. Next, a polygon was constructed around the European populations of the 1000Genomes data (CEU, FIN, GBR, IBS and TSI) to identify population outliers in our own data by considering PC1 and PC2. In more detail, the polygon was generated by first transforming the PC1:PC2-coordinates of the European individuals from 1000Genomes and of our samples into spatial data, using R package *sp* [44,45]. Ideally, a circle around each European 1000Genomes data point (sample) would represent the genetic neighborhood of the respective individual, and the union of these circles would be the region of probable European ancestry. However, that is technically difficult and therefore R package *rgeos* was used to calculate 20-polygonal approximations of circles with a width of 0.0005 around each data point [46] (Figure A1). The width of these circle-polygons was chosen such that the union of all circle-polygons was connected. The width roughly equaled 1/8 of the mean of the first PC and 1/4 of the mean of the second PC of the 1000Genomes European data. As a boundary of the union of the circle-polygons, a polygon was then computed with an additional distance of 0.0005 to the circle-polygons to smooth indentations. Finally, we gauged the samples from our data set against this boundary and every sample outside the boundary was removed.

As a second approach to remove population outliers, we applied the K nearest neighbor (KNN) method suggested in [47] using R packages *bigsnpr* and *bigparallelr* [48,49]. Utilizing a scree plot, three PCs were considered important and a threshold of 0.15 was used for the KNN statistics.

**Table A1:** Cohorts used in this study.

Cohort	N	N Cases	N Controls	N Female Cases	N Female Controls	Age-at-Sampling Cases ^1^	Age-at-Sampling Controls ^1^	Age-at-Onset Cases ^1^
Kiel PD	184	184	0	59 (32%)	0	68 [61–76]	-	58 [48–68]
Luebeck PD	928	395	533	139 (35%)	323 (61%)	68 [57–75]	44 [35–48]	60 [51–68]
EPIPARK [13]	1271	525	746	205 (39%)	353 (47%)	69 [60–76]	67 [61–71]	60 [52–70]
DeNoPa [14]	241	149	92	52 (35%)	32 (35%)	67 [59–73]	67 [62–70]	67 [59–73]
Popgen [15,16]	3754	661	3093	262 (40%)	1527 (49%)	71 [66–77]	54 [41–65]	64 [56–71]

^1^ Median and interquartile-range. PD: Parkinson’s disease.

**Table A2:** SNPs omitted from PD-PRS.

SNP Location ^1^	Beta ^2^	GS ^3^	MAF ^4^
1:1,186,833	−0.4394	no	0.0178
1:145,716,763	0.0448	no	not imputed
1:154,837,939	0.2467	no	0.0052
1:155,205,634	0.7662	yes	0.0022
1:232,161,497	−0.2638	no	0.0087
1:62,675,673	0.317	no	0.0134
2:100,906,427	0.1534	no	0.0098
2:102,368,870	0.2332	no	0.0048
2:102,655,773	0.2056	no	0.0046
2:136,388,639	−0.0656	no	0.0513
2:191,364,828	0.2497	no	0.0079
2:63,783,507	0.173	no	0.0094
3:112,245,295	−0.1391	no	0.9907
3:48,406,286	0.0789	no	0.0398
3:96,921,359	0.1607	no	0.0069
3:97,799,541	0.1819	no	0.0062
4:133,792,853	0.1797	no	0.0057
4:77,645,873	−0.2104	no	0.0096
4:90,603,678	−0.203	no	0.0087
4:90,673,143	−0.3266	no	0.0032
4:90,810,340	0.3754	no	0.0062
4:90,955,553	0.2561	no	0.0052
4:90,967,340	0.2829	no	0.0081
4:91,033,047	0.3361	no	0.0078
4:91,278,545	0.3511	no	0.0022
5:112,288,617	0.2085	no	0.0076
5:141,311,896	0.1052	no	0.0434
5:177,972,560	0.1641	no	0.0080
5:60,150,889	0.1637	no	0.0069
6:109,972,453	0.1744	no	0.0071
6:27,483,385	0.1698	no	0.0072
6:32,036,055	−0.1716	no	0.0063
6:34,800,390	−0.2314	no	0.0029
6:48,781,938	0.2449	no	0.0087
7:6,070,199	0.1652	no	0.0096
9:116,138,770	0.2529	no	0.0042
9:139,566,889	−0.0812	no	0.1093
10:102,056,734	0.3817	no	0.0019
10:103,373,463	0.1323	no	0.0099
10:103,941,875	0.1667	no	0.0080
10:105,038,008	0.1579	no	0.0076
10:27,198,118	0.2103	no	0.0012
10:48,433,720	0.0481	no	0.1562
11:93,561,149	0.1769	no	0.0041
12:123,341,500	0.2448	no	0.0064
12:123,923,612	0.2771	no	0.0077
12:40,734,202	2.4354	yes	0.0001
12:72,179,446	0.2839	no	0.0156
14:103,351,731	0.1973	no	0.0046
16:429,926	0.2396	no	0.0077
16:71,451,526	0.2423	no	0.0065
17:43,516,175	−0.2917	no	0.0130
17:43,559,955	−0.2548	no	0.0098
17:43,857,449	−0.3906	no	0.0162
17:44,687,696	−0.5875	no	0.0172
17:44,914,558	−0.1824	no	0.0095
17:44,916,533	0.2253	no	0.0095
17:8,209,654	−0.1341	no	0.0131
19:11,084,467	0.2043	no	0.0083
19:38,222,914	0.1495	no	0.0085
19:39,756,425	−0.1751	no	0.0092
20:31,687,446	0.2054	no	0.0080
median [IQR] omitted 62 SNPs	0.207 [0.166, 0.262] ^5^		0.0080 [0.0062, 0.0098]
median [IQR] 1743 SNPs used in this study	0.056 [0.042, 0.091] ^5^		0.1916 [0.0102, 0.4407]

^1^ Location of SNPs, given as chromosome:basepair position. ^2^ β from the meta-GWAS performed by Nalls et al. [2]. ^3^ Genome-wide significant (GS) in the meta-GWAS performed by Nalls et al. [2]. ^4^ MAF in our data set. ^5^ median and IQR of the absolute values of β. SNP: single nucleotide polymorphism, MAF: minor allele frequency, IQR: inter-quartile range, PRS: polygenic risk score, PD: Parkinson’s disease.

**Table A3:** Incidence of PD in different age groups.

Age Interval in Years	Incidence ^1^	Survival ^2^	Residual Lifetime Incidence ^3^
50–54	0.0002	0.994	0.017
55–59	0.0005	0.992	0.017
60–64	0.0009	0.987	0.018
65–69	0.0016	0.983	0.018
70–74	0.0034	0.974	0.018
75–79	0.0051	0.958	0.016
80–84	0.0067	0.929	0.014
85–89	0.0072	0.874	0.011
90–94	0.0056	0.782	0.007
95+	0.0052	0.654	0.005

^1^ Probability to develop PD during age interval (from [32]). ^2^ Probability to survive a year from the respective age interval (from [32]). ^3^ Probability to develop PD in later life (see Methods section). PD: Parkinson’s disease.

**Table A4:** Most relevant SNPs located within genes.

HGNC Symbol ^1^	Chr	AUC	Start ^2^	End ^3^	SNP Position ^4^	A1 ^5^	A2 ^6^	GS ^7^
ENSG00000251095	4	0.643	90,472,507	90,647,654	90,626,111	G	A	yes
*SNCA*	4	0.641	9,0645,250	90,759,466	90,684,278	A	G	no
*HIP1R*	12	0.640	123,319,000	123,347,507	123,326,598	G	T	yes
*TMEM175*	4	0.639	926,175	952,444	951,947	T	C	yes
*SNCA*	4	0.638	90,645,250	90,759,466	90,757,294	A	C	no
*ASH1L*	1	0.637	155,305,059	155,532,598	155,437,711	G	A	no
*UBQLN4*	1	0.634	156,005,092	156,023,585	156,007,988	G	A	no
ENSG00000225342	12	0.633	40,579,811	40,617,605	40,614,434	C	T	yes
*LRRK2*	12	0.633	40,590,546	40,763,087	40,614,434	C	T	yes
*STX1B*	16	0.632	31,000,577	31,021,949	31,004,169	T	C	no
*INPP5F*	10	0.631	121,485,609	121,588,652	121,536,327	G	A	yes
*CCSER1*	4	0.631	91,048,686	92,523,064	91,164,040	C	T	no
*SLC2A13*	12	0.630	40,148,823	40,499,891	40,388,109	C	T	no
*FBXL19*	16	0.630	30,934,376	30,960,104	30,943,096	A	G	no
ENSG00000251095	4	0.629	90,472,507	90,647,654	90,619,032	C	T	no
*CAB39L*	13	0.629	49,882,786	50,018,262	49,927,732	T	C	yes
*STK39*	2	0.628	168,810,530	169,104,651	168,979,290	C	T	no
*CCT3*	1	0.628	156,278,759	156,337,664	156,300,731	T	C	no
ENSG00000225342	12	0.627	40,579,811	40,617,605	40,614,656	A	G	no
*LRRK2*	12	0.627	40,590,546	40,763,087	40,614,656	A	G	no
*SH3GL2*	9	0.627	17,579,080	17,797,127	17,726,888	C	T	no
*LRRK2*	12	0.626	40,590,546	40,763,087	40,713,899	T	C	no
ENSG00000251095	4	0.625	90,472,507	90,647,654	90,573,396	G	A	no
*ASXL3*	18	0.625	31,158,579	31,331,156	31,304,318	G	T	yes
*SH3GL2*	9	0.624	17,579,080	17,797,127	17,579,690	T	G	yes
ENSG00000259675	15	0.623	61,931,548	62,007,370	61,997,385	T	C	yes
*RGS10*	10	0.623	121,259,340	121,302,220	121,260,786	A	G	no
*CASC16*	16	0.622	52,586,002	52,686,017	52,636,242	C	A	yes
*EPRS*	1	0.621	220,141,943	220,220,000	220,163,026	C	A	no
*BRIP1*	17	0.621	59,758,627	59,940,882	59,918,091	A	G	no
*PCGF3*	4	0.620	699,537	764,428	758,444	C	T	no
ENSG00000249592	4	0.620	756,175	775,637	758,444	C	T	no
ENSG00000233799	4	0.620	758,275	758,862	758,444	C	T	no
*NDUFAF2*	5	0.620	60,240,956	60,448,853	60,297,500	A	G	no
*DLG2*	11	0.619	83,166,055	85,338,966	83,488,901	C	T	no
*SEC16A*	9	0.618	139,334,549	139,372,141	139,336,813	T	G	no
*FCGR2A*	1	0.617	161,475,220	161,493,803	161,478,859	T	C	no
*SPTSSB*	3	0.617	161,062,580	161,090,668	161,077,630	A	G	yes
*DSCAM*	21	0.616	41,382,926	42,219,065	41,452,034	C	T	no
*GAK*	4	0.616	843,064	926,161	893,712	C	T	no
*CTSB*	8	0.615	11,700,033	11,726,957	11,707,174	A	G	no
*ASH1L*	1	0.615	155,305,059	155,532,598	155,347,819	A	C	no
*DCST1*	1	0.614	155,006,300	155,023,406	155,014,968	T	G	no
*LRSAM1*	9	0.614	130,213,765	130,265,780	130,261,113	G	A	no
*UBAP2*	9	0.614	33,921,691	34,048,947	34,046,391	C	T	yes
*GCH1*	14	0.613	55,308,726	55,369,570	55,348,869	C	T	yes
*PCGF2*	17	0.613	36,890,150	36,906,070	36,896,751	G	A	no
*SETD5*	3	0.612	9,439,299	9,520,924	9,504,099	G	A	no
*LRRK2*	12	0.611	40,590,546	40,763,087	40,753,796	T	C	no
*PRSS3*	9	0.611	33,750,515	33,799,230	33,778,399	G	A	no
*KANSL1*	17	0.611	44,107,282	44,302,733	44,189,067	A	G	no
ENSG00000214871	7	0.610	23,210,760	23,234,503	23,232,659	T	C	no
*NUPL2*	7	0.610	23,221,446	23,240,630	23,232,659	T	C	no
*SEC23IP*	10	0.610	121,652,223	121,702,014	121,667,020	T	C	no
ENSG00000251095	4	0.610	90,472,507	90,647,654	90,538,467	A	G	no
*SLC38A1*	12	0.609	46,576,846	46,663,800	46,623,807	G	A	no
*MED12L*	3	0.609	150,803,484	151,154,860	151,112,968	C	A	no
*NOD2*	16	0.608	50,727,514	50,766,988	50,736,656	A	G	yes
*UBTF*	17	0.608	42,282,401	42,298,994	42,294,462	A	G	no
*BTN2A2*	6	0.608	26,383,324	26,395,102	26,389,926	C	T	no
*PGS1*	17	0.607	76,374,721	76,421,195	76,377,458	A	G	no
*MRVI1*	11	0.607	10,594,638	10,715,535	10,660,840	G	T	no
*TMEM163*	2	0.607	135,213,330	135,476,570	135,443,940	A	G	no
ENSG00000264031	17	0.606	27,887,565	28,034,108	27,897,585	T	C	no
*TP53I13*	17	0.606	27,893,070	27,900,175	27,897,585	T	C	no
*ZNF165*	6	0.606	28,048,753	28,057,341	28,054,198	A	G	no
*PCGF3*	4	0.606	699,537	764,428	733,630	G	A	no
*PITPNM2*	12	0.605	123,468,027	123,634,562	123,585,705	C	T	no
*PCGF3*	4	0.605	699,537	764,428	734,351	A	G	no
*C10orf32-ASMT*	10	0.605	104,614,029	104,661,656	104,635,103	G	A	no
*AS3MT*	10	0.605	104,629,273	104,661,656	104,635,103	G	A	no
ENSG00000232667	7	0.604	79,959,508	80,014,295	79,998,372	T	C	no
*RNF141*	11	0.604	10,533,225	10,562,777	10,558,777	A	G	yes
*STK39*	2	0.604	168,810,530	169,104,651	169,023,263	T	C	no
*CCSER1*	4	0.603	91,048,686	92,523,064	91,057,794	A	G	no
*SEZ6L2*	16	0.602	29,882,480	29,910,868	29,892,184	G	A	no
*VSTM5*	11	0.602	93,551,398	93,583,697	93,576,556	T	C	no
*SPATA19*	11	0.602	133,710,526	133,715,433	133,714,560	A	C	no
ENSG00000251095	4	0.601	90,472,507	90,647,654	90,606,518	T	G	no
*H2AFX*	11	0.600	118,964,564	118,966,177	118,965,479	G	A	no
*MSTO1*	1	0.599	155,579,979	155,718,153	155,698,425	C	T	no
*MSTO2P*	1	0.599	155,581,011	155,720,105	155,698,425	C	T	no
*DAP3*	1	0.599	155,657,751	155,708,801	155,698,425	C	T	no
*GABRB1*	4	0.599	46,995,740	47,428,461	47,372,139	A	C	no
*TMEM163*	2	0.599	135,213,330	135,476,570	135,464,616	A	G	yes
*MFSD6*	2	0.598	191,273,081	191,373,931	191,300,402	A	G	no
*AMPD3*	11	0.598	10,329,860	10,529,126	10,525,791	A	C	no
*ADD1*	4	0.598	2,845,584	2,931,803	2,901,349	A	G	no
*NSF*	17	0.597	44,668,035	44,834,830	44,808,902	G	A	no
*HCAR1*	12	0.597	123,104,824	123,215,390	123,124,138	T	C	no
*NR1I3*	1	0.597	161,199,456	161,208,092	161,205,966	G	T	no
*GAK*	4	0.596	843,064	926,161	903,249	G	A	no
*EIF3K*	19	0.595	39,109,735	39,127,595	39,116,961	A	G	no
*BPTF*	17	0.595	65,821,640	65,980,494	65,885,911	C	T	no
*FBRSL1*	12	0.595	133,066,137	133,161,774	133,081,895	C	T	no
ENSG00000260958	16	0.594	34,442,308	34,518,517	34,466,252	T	C	no
*RIT2*	18	0.594	40,323,192	40,695,657	40,673,380	A	G	yes
*C10orf2*	10	0.594	102,747,124	102,754,158	102,747,363	G	T	no
*MYOC*	1	0.593	171,604,557	171,621,823	171,612,267	G	A	no
*XPO1*	2	0.592	61,704,984	61,765,761	61,763,207	T	C	no
*CRHR1*	17	0.591	43,699,267	43,913,194	43,744,203	C	T	yes
ENSG00000263715	17	0.591	43,699,274	43,893,909	43,744,203	C	T	yes
*PPP6R2*	22	0.590	50,781,733	50,883,514	50,794,282	C	A	no
*NRG1*	8	0.590	31,496,902	32,622,548	31,942,557	G	A	no
*NRG1-IT1*	8	0.590	31,883,735	31,996,991	31,942,557	G	A	no
*LTK*	15	0.590	41,795,836	41,806,085	41,798,614	T	C	no
*SAA1*	11	0.589	18,287,721	18,291,524	18,290,067	G	T	no
*KCNIP3*	2	0.589	95,963,052	96,051,825	96,025,765	A	G	no
*PCGF3*	4	0.588	699,537	764,428	749,620	T	G	no
*ART3*	4	0.588	76,932,337	77,033,955	76,990,450	C	T	no
*ARL15*	5	0.588	53,179,775	53,606,412	53,537,742	G	A	no
ENSG00000272414	4	0.587	77,135,193	77,204,933	77,198,054	C	T	yes
*FAM47E*	4	0.587	77,172,874	77,232,282	77,198,054	C	T	yes
*FAM47E-STBD1*	4	0.587	77,172,886	77,232,752	77,198,054	C	T	yes
*SCARB2*	4	0.587	77,079,890	77,135,046	77,100,807	T	C	no
*WNT3*	17	0.587	44,839,872	44,910,520	44,868,187	G	A	no
*DSCR9*	21	0.586	38,580,804	38,594,037	38,593,620	G	T	no
*MYLK3*	16	0.586	46,740,891	46,824,319	46,778,070	G	A	no
ENSG00000251095	4	0.586	90,472,507	90,647,654	90,513,701	G	A	no
*BST1*	4	0.585	15,704,573	15,739,936	15,737,348	G	A	yes
*C9orf129*	9	0.585	96,080,481	96,108,696	96,087,807	C	T	no
*MMRN1*	4	0.584	90,800,683	90,875,780	90,804,532	C	T	no
*MAPT-AS1*	17	0.584	43,921,017	43,972,966	43,935,838	T	C	no
*MCCC1*	3	0.584	182,733,006	182,833,863	182,760,073	T	G	yes
*MUC19*	12	0.583	40,787,197	40,964,632	40,829,565	G	A	no
ENSG00000258167	12	0.583	40,789,655	40,837,649	40,829,565	G	A	no
*CCNT2-AS1*	2	0.583	135,493,034	135,676,280	135,500,179	G	A	no
*XKR6*	8	0.583	10,753,555	11,058,875	10,999,583	C	T	no
*RCAN2*	6	0.582	46,188,475	46,459,709	46,229,444	C	T	no
*ITGA8*	10	0.582	15,555,948	15,762,124	15,563,450	C	T	no
*RANBP9*	6	0.581	13,621,730	13,711,796	13,657,040	G	A	no
*IGF2BP3*	7	0.581	23,349,828	23,510,086	23,462,162	C	A	no
*FAM47E*	4	0.580	77,135,193	77,204,933	77,202,861	A	G	no
ENSG00000272414	4	0.580	77,172,874	77,232,282	77,202,861	A	G	no
*FAM47E-STBD1*	4	0.580	77,172,886	77,232,752	77,202,861	A	G	no
ENSG00000251095	4	0.579	90,472,507	90,647,654	90,594,987	G	A	no
*SCARB2*	4	0.578	77,079,890	77,135,046	77,111,032	C	T	no
*ARHGAP27*	17	0.578	43,471,275	43,511,787	43,472,507	A	G	no
*ZYG11B*	1	0.578	53,192,126	53,293,014	53,233,374	T	C	no
ENSG00000244128	3	0.577	164,924,748	165,373,211	165,020,212	A	G	no
*PER1*	17	0.577	8,043,790	8,059,824	8,051,639	A	G	no
*KCNS3*	2	0.577	18,059,114	18,542,882	18,132,092	C	T	no
*HIBCH*	2	0.576	191,054,461	191,208,919	191,071,057	G	A	no
*RN7SL416P*	7	0.576	100,127,987	100,128,282	100,128,114	G	A	no
*YLPM1*	14	0.575	75,230,069	75,322,244	75,234,329	G	A	no
*FGFRL1*	4	0.574	1,003,724	1,020,685	1,008,212	C	T	no
*CRHR1*	17	0.574	43,699,267	43,913,194	43,798,308	G	A	yes
ENSG00000263715	17	0.574	43,699,274	43,893,909	43,798,308	G	A	yes
*HIP1R*	12	0.574	123,319,000	123,347,507	123,334,442	C	T	no
*MYO15B*	17	0.573	73,584,139	73,622,929	73,587,257	A	G	no
*PITPNM2*	12	0.573	123,468,027	123,634,562	123,525,280	A	G	no
*PREX2*	8	0.573	68,864,353	69,149,265	69,029,244	C	A	no
ENSG00000255468	11	0.573	66,115,421	66,132,275	66,115,782	G	T	no
*SIPA1L2*	1	0.572	232,533,711	232,697,304	232,664,611	C	T	yes
*AMPD3*	11	0.571	10,329,860	10,529,126	10,475,856	G	A	no
*PAM*	5	0.571	102,089,685	102,366,809	102,363,402	C	T	no
*IFT140*	16	0.571	1,560,428	1,662,111	1,593,645	C	T	no
*TMEM204*	16	0.571	1,578,689	1,605,581	1,593,645	C	T	no
*CLIP1*	12	0.570	122,755,979	122,907,179	122,891,863	C	T	no
*ABCB9*	12	0.570	123,405,498	123,466,196	123,418,656	G	T	no
*ZC3H7B*	22	0.570	41,697,526	41,756,151	41,755,105	A	G	no
*CRHR1*	17	0.569	43,699,267	43,913,194	43,784,228	T	C	no
ENSG00000263715	17	0.569	43,699,274	43,893,909	43,784,228	T	C	no
*LRRK2*	12	0.569	40,590,546	40,763,087	40,730,463	C	T	no
ENSG00000235423	12	0.569	123,736,577	123,746,030	123,744,082	C	A	no
*MSRA*	8	0.568	9,911,778	10,286,401	10,280,818	A	C	no
*LYVE1*	11	0.568	10,578,513	10,633,236	10,628,883	G	A	no
*MRVI1*	11	0.568	10,594,638	10,715,535	10,628,883	G	A	no
*FAM162A*	3	0.568	122,103,023	122,131,181	122,109,601	T	C	no
*MMRN1*	4	0.567	90,800,683	90,875,780	90,868,355	T	C	no
ENSG00000236656	1	0.567	158,444,244	158,464,676	158,453,419	A	C	no
ENSG00000235495	2	0.567	67,792,736	67,911,209	67,806,472	A	G	no
*DEFB119*	20	0.566	29,964,967	29,978,406	29,971,435	G	A	no
*NGEF*	2	0.566	233,743,396	233,877,982	233,864,457	C	T	no
*MGAT5*	2	0.566	134,877,554	135,212,192	135,202,455	A	G	no
*ASAH1*	8	0.565	17,913,934	17,942,494	17,927,609	C	T	no
*CPNE8*	12	0.565	39,040,624	39,301,232	39,174,139	T	G	no
*SEMA3G*	3	0.565	52,467,069	52,479,101	52,468,940	T	C	no
*PBRM1*	3	0.564	52,579,368	52,719,933	52,649,748	A	G	no
*HMBOX1*	8	0.564	28,747,911	28922281	28,809,951	A	G	no
*HMBOX1-IT1*	8	0.564	28,807,193	28,813,472	28,809,951	A	G	no
*SNCA*	4	0.563	90,645,250	90,759,466	90,700,329	T	C	no
*MAPT*	17	0.563	43,971,748	44,105,700	44,071,851	G	A	no
ENSG00000258881	2	0.563	71,166,448	71,222,466	71,202,989	T	C	no
ENSG00000251095	4	0.562	90,472,507	90,647,654	90,627,967	G	A	no
*CRHR1*	17	0.562	43,699,267	43,913,194	43,901,665	T	C	no
*ARHGEF7*	13	0.562	111,766,906	111,958,084	111,863,720	C	T	no
*GNPTAB*	12	0.561	102,139,275	102,224,716	102,151,977	C	T	no
*FAM220A*	7	0.561	6,369,040	6,388,612	6,369,946	A	G	no
*BRD2*	6	0.561	32,936,437	32,949,282	32,941,506	C	T	no
*ATG4D*	19	0.561	10,654,571	10,664,094	10,663,997	C	T	no
*KRI1*	19	0.561	10,663,761	10,676,713	10,663,997	C	T	no
*FBXO34*	14	0.560	55,738,021	55,828,636	55,801,687	A	C	no
ENSG00000258455	14	0.560	55,792,552	55,806,219	55,801,687	A	C	no
*CCDC101*	16	0.560	28,565,236	28,603,111	28,566,158	G	T	no
*C14orf159*	14	0.560	91,526,677	91,691,976	91,682,844	T	C	no
*KIF21A*	12	0.560	39,687,030	39,837,192	39,738,666	G	A	no
*PRRC2C*	1	0.559	171,454,651	171,562,650	171,471,672	T	C	no
*RNF141*	11	0.559	10,533,225	10,562,777	10,560,447	A	C	no
*SOX2-OT*	3	0.559	180,707,558	181,554,668	180,797,921	T	G	no
*SLC2A13*	12	0.558	40,148,823	40,499,891	40,437,969	A	G	no
*RPP14*	3	0.558	58,291,974	58,310,422	58,292,485	G	A	no
*DGKG*	3	0.557	185,823,457	186,080,026	185,834,290	T	C	no
ENSG00000251364	11	0.557	7,448,497	7,533,746	7,532,175	T	G	no
*OLFML1*	11	0.557	7,506,619	7,532,608	7,532,175	T	G	no
*ADAM15*	1	0.557	155,023,042	155,035,252	155,033,317	T	C	no
*TRHDE*	12	0.556	72,481,046	73,059,422	72,714,601	G	T	no
*GAK*	4	0.556	843,064	926,161	852,939	G	A	no
*CCDC134*	22	0.555	42,196,683	42,222,303	42,216,326	A	G	no
*LZTS2*	10	0.555	10,275,6375	102,767,593	102,764,511	G	A	no
*SLC44A2*	19	0.555	10,713,133	10,755,235	10,730,352	G	A	no
*FYN*	6	0.554	111,981,535	112,194,655	112,164,313	G	A	no
*RNF212*	4	0.554	1,050,038	1,107,350	1,082,829	T	C	no
*CCSER1*	4	0.553	91,048,686	92,523,064	91,383,333	G	A	no
*ZNF589*	3	0.553	48,282,590	48,340,743	48,333,546	T	C	no
*FGF14*	13	0.553	102,372,134	103,054,124	102,996,713	A	G	no
*FGF14-IT1*	13	0.553	102,944,677	103,046,869	102,996,713	A	G	no
*TFRC*	3	0.552	195,754,054	195,809,060	195,775,449	C	T	no
*MAEA*	4	0.552	1,283,639	1,333,935	1,312,394	C	T	no
*ANKRD11*	16	0.551	89,334,038	89,556,969	89,369,869	A	G	no
*ZZZ3*	1	0.551	78,028,101	78,149,104	78,070,458	C	T	no
*DNM3*	1	0.551	171,810,621	172,387,606	171,845,192	G	T	no
*LARP1B*	4	0.550	128,982,423	129,144,086	129,107,049	T	C	no
*STK39*	2	0.550	168,810,530	169,104,651	169,071,190	G	T	no
*NEXN*	1	0.550	78,354,198	78,409,580	78,392,446	G	A	no
*CD38*	4	0.550	15,779,898	15,854,853	15,829,612	A	G	no
*HAVCR1*	5	0.549	156,456,424	156,486,130	156,479,424	A	C	no
*SCAND3*	6	0.549	28,539,407	28,583,989	28,547,283	T	C	no
*APOM*	6	0.548	31,620,193	31,625,987	31,622,606	C	A	no
*TRIM37*	17	0.548	57,059,999	57,184,282	57,111,269	A	C	no
*OR9Q1*	11	0.548	57,791,353	57,949,088	57,870,219	G	A	no
*KIAA1841*	2	0.547	61,293,006	61,391,960	61,347,469	C	T	no
*TATDN2*	3	0.547	10,289,707	10,322,902	10,300,941	A	G	no
ENSG00000272410	3	0.547	10,291,056	10,327,480	10,300,941	A	G	no
*ZNF320*	19	0.547	53,367,043	53,400,946	53,399,832	C	T	no
ENSG00000272657	21	0.546	35,445,892	35,732,332	35,677,897	G	A	no
ENSG00000214955	21	0.546	35,577,356	35,697,334	35,677,897	G	A	no
*ITGAL*	16	0.546	30,483,979	30,534,506	30,520,856	C	T	no
*UNKL*	16	0.546	1,413,206	1,464,752	1,436,510	G	A	no
*FYN*	6	0.545	111,981,535	112,194,655	112,122,373	C	T	no
*SYBU*	8	0.545	110,586,207	110,704,020	110,644,774	T	C	no
*AGMO*	7	0.545	15,239,943	15,601,640	15,262,499	G	T	no
*MED12L*	3	0.544	150,803,484	151,154,860	151,133,211	G	A	no
*SYNDIG1*	20	0.544	24,449,835	24,647,252	24,645,939	G	A	no
*MYO7A*	11	0.544	76,839,310	76,926,284	76,920,983	A	G	no
*CAPRIN2*	12	0.543	30,862,486	30,907,885	30,895,251	T	C	no
*BRSK2*	11	0.543	1,411,129	1,483,919	1,478,565	T	C	no
*ARID2*	12	0.542	46,123,448	46,301,823	46,134,812	T	C	no
*RALYL*	8	0.542	85,095,022	85,834,079	85,772,129	A	G	no
*HCAR1*	12	0.542	123,104,824	123,215,390	123,189,794	T	C	no
ENSG00000256249	12	0.542	123,171,672	123,200,526	123,189,794	T	C	no
*SPPL2B*	19	0.541	2,328,614	2,355,099	2,341,047	C	T	yes
*RNF165*	18	0.541	43,906,772	44,043,103	44,040,660	T	C	no
*HSF5*	17	0.541	56,497,528	56,565,745	56,507,063	C	T	no
*ENO3*	17	0.540	4,851,387	4,860,426	4,858,206	A	G	no
*WBP1L*	10	0.539	104,503,727	104,576,021	104,562,212	C	T	no
*ERC2*	3	0.538	55,542,336	56,502,391	56,014,781	A	G	no
*MYO1H*	12	0.538	109,785,708	109,893,328	109,846,466	G	T	no
*MAEA*	4	0.538	1,283,639	1,333,935	1,311,933	G	T	no
ENSG00000244036	7	0.538	129,593,074	129,666,391	129,663,496	C	T	no
*ZC3HC1*	7	0.538	129,658,126	129,691,291	129,663,496	C	T	no
*CSMD1*	8	0.537	2,792,875	4,852,494	3,078,351	A	G	no
ENSG00000259848	2	0.537	95,533,231	95,613,086	95,555,581	T	C	no
*POU2F3*	11	0.536	120,107,349	120,190,653	120,178,753	T	G	no
*HLA-DOA*	6	0.536	32,971,955	32,977,389	32,973,303	T	C	no
*TMPO*	12	0.536	98,909,290	98,944,157	98,939,838	C	A	no
*MTF2*	1	0.536	93,544,792	93,604,638	93,570,368	G	A	no
*SLC16A10*	6	0.535	111,408,781	111,552,397	111,489,059	G	T	no
ENSG00000250003	5	0.535	38,025,799	38,184,034	38,046,354	G	A	no
ENSG00000225981	7	0.534	1,499,573	1,503,644	1,502,497	C	T	no
*LRRK2*	12	0.534	4,059,0546	40,763,087	40,707,861	C	T	no
*TRAPPC13*	5	0.533	64,920,543	64,962,060	64,952,500	C	T	no
*METTL13*	1	0.533	171,750,788	171,783,163	171,772,453	T	G	no
ENSG00000259675	15	0.533	61,931,548	62,007,370	62,005,917	C	A	no
*AIRE*	21	0.532	45,705,721	45,718,531	45,708,277	C	T	no
ENSG00000272305	3	0.532	53,003,135	53,133,469	53,087,621	A	G	no
*C6orf10*	6	0.531	32,256,303	32,339,684	32,303,848	G	A	no
*HLA-DQA2*	6	0.530	32,709,119	32,714,992	32,712,666	C	T	no
*XPO1*	2	0.530	61,704,984	61,765,761	61,763,170	C	T	no
*HLA-DQB1*	6	0.529	32,627,244	32,636,160	32,634,646	T	C	no
*LRRK2*	12	0.529	40,579,811	40,617,605	40,607,566	G	A	no
ENSG00000225342	12	0.529	40,590,546	40,763,087	40,607,566	G	A	no
*C1orf167*	1	0.529	11,821,844	11,849,642	11,827,776	A	G	no
ENSG00000249988	4	0.528	14,166,079	14,244,437	14,167,196	A	G	no
*LAMA2*	6	0.528	129,204,342	129,837,714	129,537,858	G	A	no
*SOX6*	11	0.528	15,987,995	16,761,138	16,158,420	G	A	no
*CCDC69*	5	0.527	150,560,613	150,603,706	150,566,196	C	T	no
ENSG00000223343	3	0.527	49,022,482	49,027,421	49,025,101	A	C	no
*MAP4K4*	2	0.527	102,313,312	102,511,149	102,468,624	A	G	no
*KLHL7*	7	0.526	23,145,353	23,217,533	23,208,043	G	A	no
ENSG00000253194	6	0.526	119,255,950	119,352,706	119,322,992	C	T	no
*FAM184A*	6	0.526	119,280,928	119,470,552	119,322,992	C	T	no
*QRICH1*	3	0.525	49,067,140	49,131,796	49,083,566	G	A	no
*SYT17*	16	0.525	19,179,293	19,279,652	19,279,380	T	C	no
*CCDC62*	12	0.524	123,258,874	123,312,075	123,296,204	G	A	no
*SHC4*	15	0.524	49,115,932	49,255,641	49,174,661	C	T	no
*PNKD*	2	0.523	219,135,115	219,211,516	219,142,491	C	T	no
*TMBIM1*	2	0.523	219,138,915	219,157,309	219,142,491	C	T	no
*DIP2C*	10	0.523	320,130	735,683	570,172	T	C	no
*SCCPDH*	1	0.523	246,887,349	246,931,439	246,893,948	C	T	no
*IP6K1*	3	0.522	49,761,727	49,823,975	49,808,007	A	G	no
*FAM167A*	8	0.522	11,278,972	11,332,224	11,309,780	G	A	no
*ADCY5*	3	0.521	123,001,143	123,168,605	123,143,272	G	A	no
*PCGF3*	4	0.521	699,537	764,428	701,896	A	G	no
*RPRD2*	1	0.520	150,335,567	150,449,042	150,438,362	A	C	no
*CARM1*	19	0.520	10,982,189	11,033,453	11,025,817	G	A	no
ENSG00000251246	1	0.519	155,036,224	155,059,283	155,055,863	G	A	no
*EFNA3*	1	0.519	155,036,224	155,060,014	155,055,863	G	A	no
*MMS22L*	6	0.519	97,590,037	97,731,093	97,662,784	G	A	no
*C12orf40*	12	0.519	40,019,969	40,302,102	40,042,940	C	T	no
*C3orf84*	3	0.518	49,215,065	49,229,291	49,220,504	A	C	no
*MMRN1*	4	0.518	90,800,683	90,875,780	90,859,279	G	A	no
*RILPL2*	12	0.517	123,899,936	123,921,264	123,912,213	T	C	no
*CHAT*	10	0.517	50,817,141	50,901,925	50,821,191	G	T	no
*TMEM161B*	5	0.517	87,485,450	87,565,293	87,513,775	C	T	no
*BIN3*	8	0.517	22,477,931	22,526,661	22,525,980	T	C	yes
*TRPM4*	19	0.516	49,660,998	49,715,093	49,695,007	A	G	no
*USP8*	15	0.516	50,716,577	50,793,280	50,741,068	A	C	no
*BCAR3*	1	0.516	94,027,347	94,312,706	94,038,847	G	A	no
*TNXB*	6	0.516	32,008,931	32,083,111	32,062,687	G	A	no

^1^ HGNC symbol or Ensemble gene ID if there is no HGNC symbol available. ^2^ Base pair position of start of gene. ^3^ Base pair position of end of gene. ^4^ Genomic position of SNP. ^5^ Major SNP allele. ^6^ Minor SNP allele. ^7^ Genome-wide significant in the meta-GWAS by Nalls et al. [2]. HGNC: HUGO Gene Nomenclature Committee, Chr: Chromosome, AUC: area under ROC curve, ROC: receiver operating characteristic, PRS: polygenic risk score, PD: Parkinson’s disease, n.a.: not available.
